# Man With an Itchy Back

**DOI:** 10.1016/j.acepjo.2024.100032

**Published:** 2025-01-13

**Authors:** Katherine Schaffer, Nevin Adamski, Brittany Ambrose, Kathleen Anderson

**Affiliations:** 1 Department of Emergency Medicine, Macon & Joan Brock Virginia Health Sciences at Old Dominion University, Norfolk, Virginia, USA; 2 Emergency Physicians of Tidewater, Norfolk, Virginia, USA

**Keywords:** global health, travel, dermatology, furuncular myiasis, Cordylobia anthropophaga, tropical medicine, incision and drainage

## 1 Case

**Figure 1:**
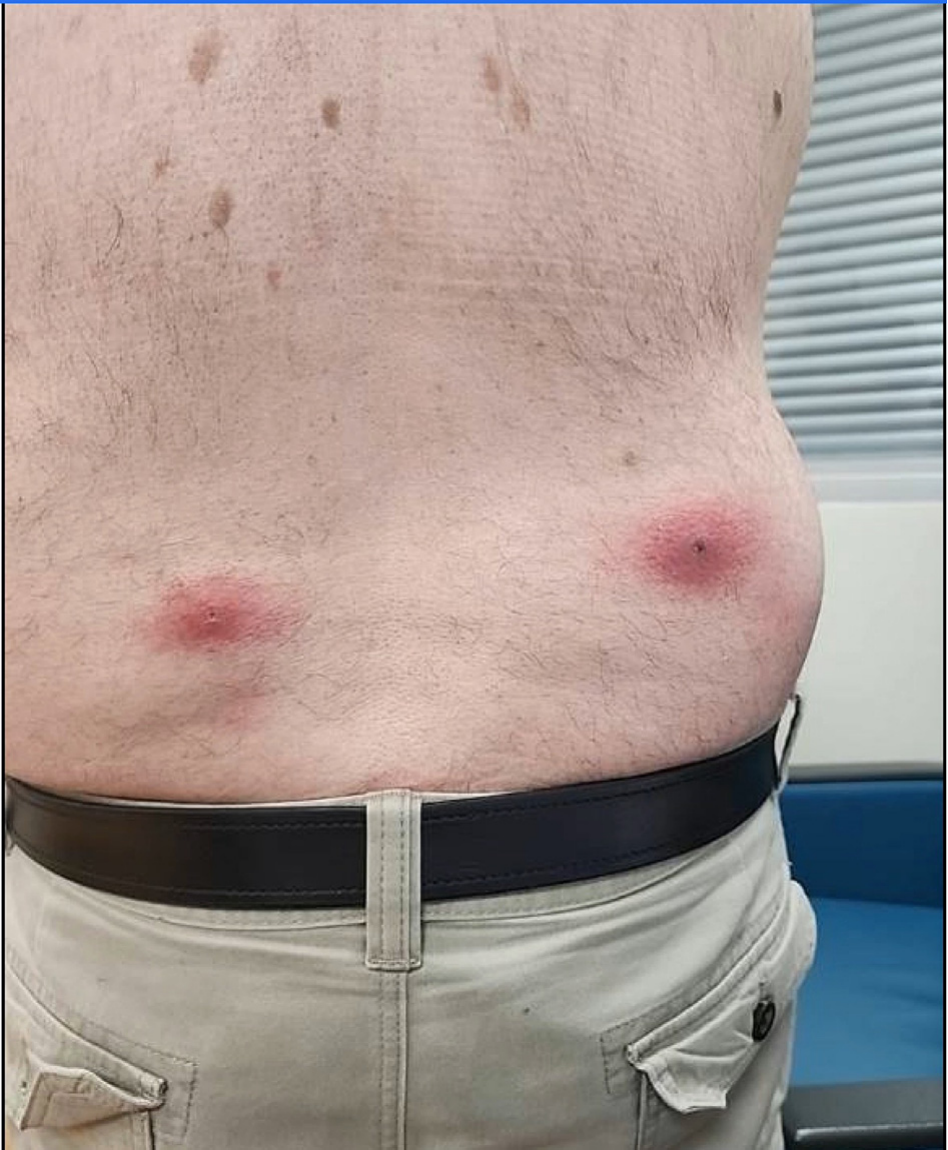
Image of the patient's back with 2 furuncular myiasis.

A 61-year-old man with no significant past medical history presented with painful bug bites on his lower back 1 day after a 1.5-week-long visit to Africa. He noticed bug bites on his lower back 3 days ago; since then, they have increased in size and become more painful. He was prescribed doxycycline at urgent care earlier that morning and had taken 1 dose. He is taking prophylactic medications for malaria and has had no fever at home. Vital signs, including temperature, are within normal limits. A physical examination shows 2 exquisitely tender papular lesions with a central punctum and surrounding erythema in the bilateral paralumbar regions (Fig 1).

**Figure 2:**
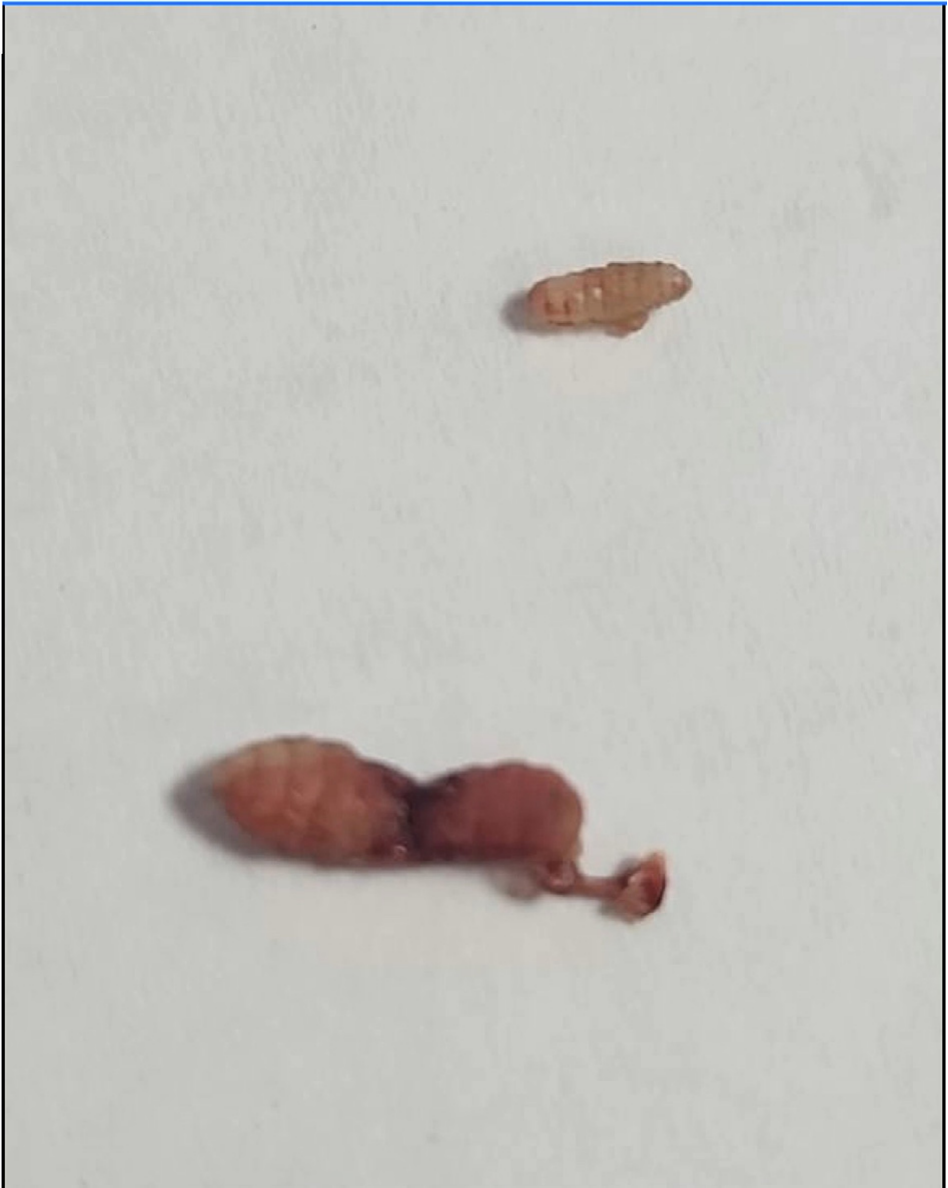
Image of larvae of *Cordylobia anthropophaga*.

Incision and drainage of the skin lesions were performed using local anesthesia. A foreign body was noted to be protruding through the skin opening during anesthetic injection on both sides. The skin openings were extended, and hemostats were used to extract the foreign bodies, which were identified as maggots (Fig 2).

## 2 Diagnosis

*Cordylobia anthropophaga.* Tumbu fly, or mango fly, is endemic in tropical Africa and causes furuncular myiasis.^1^ The female fly lays its eggs in soil or on wet clothes, and the primary larvae emerge approximately 2 days later. These larvae can then penetrate unbroken human skin and develop into tertiary larvae within the dermis.^2^ They will maintain a breathing pore in the skin from which they emerge when fully mature.

Diagnosis is based on the presence of the lesion, subjective sensation of movement, and recent travel history. Covering the punctum with gel can asphyxiate the larvae and force them to the surface. Surgical extraction may also be necessary, as in this case. Bedside ultrasonography may aid in diagnosis.

## Funding and Support

By *JACEP Open* policy, all authors are required to disclose any and all commercial, financial, and other relationships in any way related to the subject of this article as per ICMJE conflict of interest guidelines (see www.icmje.org). The authors have stated that no such relationships exist.
